# Amniotic fluid embolism

**DOI:** 10.4103/0972-5229.58537

**Published:** 2009

**Authors:** A. Rudra, S. Chatterjee, S. Sengupta, B. Nandi, J. Mitra

**Affiliations:** **From:** Department of Anaesthesiology, K.P.C. Medical College, Kolkata, India; 1 Department of Anaesthesiology, Medical College & Hospital, Kolkata, India; 2 Department of Anaesthesiology, Apollo *Gleneagles* Hospital, Kolkata, India

**Keywords:** Amniotic fluid embolism, coagulopathy, hypoxia

## Abstract

The disastrous entry of amniotic fluid into the maternal circulation leads to dramatic sequelae of clinical events, characteristically referred to as Amniotic fluid embolism (AFE). The underlying mechanism for AFE is still poorly understood. Unfortunately, this situation has very grave maternal and fetal consequences. AFE can occur during labor, caesarean section, dilatation and evacuation or in the immediate postpartum period. The pathophysiology is believed to be immune mediated which affects the respiratory, cardiovascular, neurological and hematological systems. Undetected and untreated it culminates into fulminant pulmonary edema, intractable convulsions, disseminated intravascular coagulation (DIC), malignant arrhythmias and cardiac arrest. Definite diagnosis can be confirmed by identification of lanugo, fetal hair and fetal squamous cells (squames) in blood aspirated from the right ventricle. Usually the diagnosis is made clinically and by exclusion of other causes. The cornerstone of management is a multidisciplinary approach with supportive treatment of failing organs systems. Despite improved modalities for diagnosing AFE, and better intensive care support facilities, the mortality is still high.

## Introduction

Despite its rare occurrence, the syndrome of amniotic fluid embolism (AFE) is well-known to anesthesiologists. Given its sudden and dramatic presentation and its often devastating consequences, practitioners given the responsibility of caring for an unfortunate woman with AFE remember the experience in great detail for a long time thereafter. AFE is initiated by entry of amniotic fluid into the maternal circulation and is characterized by the sudden onset of severe dyspnea, tachypnea, and cyanosis during labor, delivery or early puerperium, the mechanism of which is unclear. In addition, elements of amniotic fluid have been isolated in blood and sputum of pregnant women who did not have clinical evidence of AFE. In some women AFE may lead to mild degree of organ dysfunction while in others it may lead to coagulopathy, cardiovascular collapse, and death. Understanding its etiology and pathophysiology is incomplete, and the criteria used to make its diagnosis are controversial. Furthermore, despite advances in the care of critically ill patients, no management interventions have been found to improve the survival or long-term outcome of woman with AFE.

We have searched PubMed using keywords like amniotic fluid, amniotic fluid embolism, embolism, catastrophe, disseminated intravascular coagulation, hypoxia, and pregnancy either alone or in combination for reviewing this topic.

## Historied Aspects

Amniotic fluid embolism was first reported by Ricardo Meyer in 1926.[1] It was reported again in an experiment on laboratory animals by Warden in 1927.[2] The importance of this condition and these early studies was not established until 1941, when Steiner and Lushbaugh reported the clinical and pathological findings of 42 women who died suddenly during or just after labor.[3] The histopathology of the pulmonary vasculature of these women included mucin, amorphous eosinophilic material, and squamous cells. These findings formed the “classic” pathologic findings in AFE.

Although historically the pathophysiology was thought to involve embolization of amniotic fluid, there has been growing evidence that the syndrome results from bronchial mediators that are released after embolization occurs. Clarke *et al.*,[4,5] have proposed renaming the syndrome “anaphylactoid syndrome of pregnancy” because of the various humoral and immunologic factors implicated. Because AFE is so uncommon, no single institution has sufficient experience to assess risk factors, determine the pathophysiology and clinical course, or evaluate management strategies. Clarke *et al.*,[4] in the United States, and Tuffnell[6] in the United Kingdom have established a national registry for suspected AFE.

Entry criteria consist of the presence of the following four factors:

1. Acute hypotension or cardiac arrest
2. Acute hypoxia
3. Coagulopathy or severe clinical hemorrhage in the absence of other explanations
4. All of these occurring during labor, caesarean delivery, or dilation and evacuation or within 30 minutes postpartum with no other explanation for the clinical findings.

## Epidemiology and Risk Factors

The incidence of AFE has been reported to range from 1 in 8000 to 1 in 80,000 deliveries.[7] The syndrome typically occurs during labor, soon after vaginal or caesarean delivery, or during second-trimester dilation and evacuation procedures. In the national registry, 70% of the cases occurred during labor, 19% were recorded during caesarean delivery, and 11% occurred after vaginal delivery.[4] All of the cases noted during caesarean section had their onset soon after delivery of the infant.

A factor that is consistently related to the occurrence of AFE is a tear in the fetal membranes. Of the women included in the national registry, 78% had ruptured membranes. Two thirds of the women had artificial rupture and one third had spontaneous rupture. Placental abruption was confirmed in 13% of women.[8] These findings suggest that certain conditions may permit exposure of fetal tissue to the maternal vasculature and may increase the risk for AFE.[9]

The overall maternal mortality rate in Clarke's registry[4] was 61%, accounting for approximately 10% of all maternal deaths in the United States and resulting in permanent neurologic deficit in up to 85% of survivors.[2] The UK registry reports a case fatality rate of 37% with 7% of survivors remaining neurologically impaired.[6] These figures are lower than those reported by Morgan and Clarke and may be due to early diagnosis and advances in intensive care.

Fetal outcome is poor if the onset of AFE is before delivery while the fetus is alive in utero. Clarke *et al.*,[4] reported 79% of fetuses survived but only 50% of these were neurologically normal. In the UK registry 87% of the fetuses survived, with 29% of survivors developing hypoxic ischemic encephalopathy.[6]

## Pathophysiology

**Figure 1:**
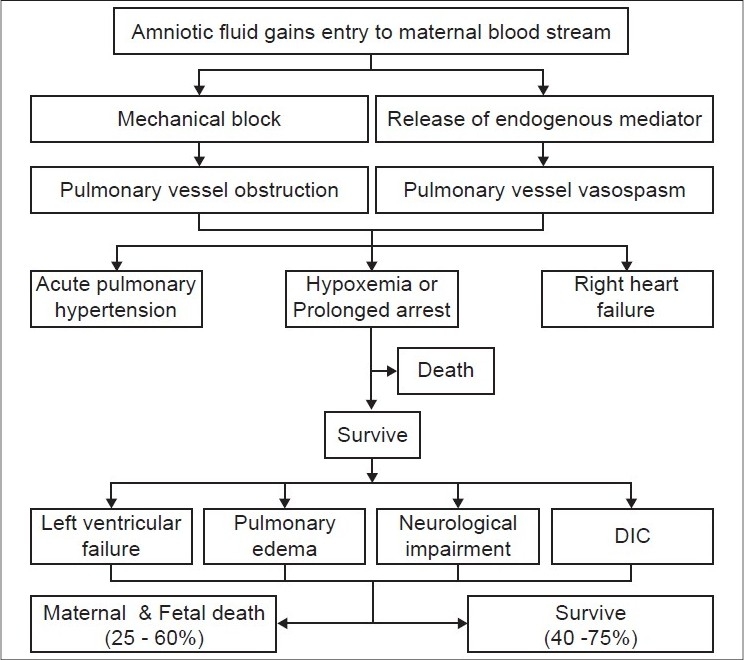
Postulated mechanism for the pathogenesis of amniotic fluid embolism; DIC - Disseminated intravascular coagulation

The pathophysiology of AFE is speculative, various theories have been published. In 1995, Clarke[4] suggested that the syndrome arose from an immune rather than embolic process. Amniotic fluid embolism is caused by fetal antigens in the amniotic fluid stimulating a cascade of endogenous immune mediators, producing a reaction similar to anaphylaxis. Biochemical mediators found in the amniotic fluid are thought to trigger the main features of anaphylactoid reaction with multisystem involvement. The fetal products found in suspension in the amniotic fluid are responsible only for the minor effects caused by the actual mechanical obstruction. Clarke[4] suggested that the syndrome of acute peripartum hypoxia, hemodynamic collapse, and coagulopathy should be described as anaphylactoid syndrome of pregnancy and not AFE. There are also striking similarities between clinical and hemodynamic findings in AFE and septic shock, which suggests a common pathological mechanism. Gei and Hankins[9] proposed a pathophysiological course [Figure 1].

The initial respiratory reaction possibly begins with transient pulmonary vasospasm.[10] Although this possible transient vasospasm has not been documented (probably because the signs and symptoms appear so abruptly in a seemingly healthy person who is not monitored via invasive methods), vasospasm may be caused by amniotic microemboli that trigger the release of arachidonic acid metabolites,[10] and leads to pulmonary hypertension, intrapulmonary shunting, bronchoconstriction, and severe hypoxia.[11] Exactly which components of amniotic fluid actually cause this effect is unknown, but Clarke[10] suggested that abnormal components such as meconium may play a role. Many experts[11–16] speculate that maternal mediators also may have an influence.

The second manifestation includes negative inotropism and left ventricular failure resulting in increasing pulmonary edema and hypotension quickly leading to shock.

The third manifestation is a neurological response to the respiratory and hemodynamic injury, which may include seizures, confusion, or coma.[9]

About 40% to 50% of patients who survive to this point have severe coagulopathy, usually disseminated intravascular coagulation, which results in uncontrollable uterine bleeding along with bleeding from puncture sites such as insertion sites for intravenous and epidural catheters.[9] This coagulopathy is thought to be precipitated by several procoagulant components of amniotic fluid, most notably thromboplastin, which initiates the extrinsic pathway of the clotting cascade and results in excessive fibrinolytic activity.[9]

Depending upon the magnitude of the events and the maternal physiological reserve, the patient may not recover from the injury. During this convalescent period, patient may die as a consequence of the severe lung or brain injury, multi-organ failure, or from an infection acquired in the intensive care unit.[10]

## Clinical Presentation

Amniotic fluid embolism typically occurs during labor and delivery[17] or in the immediate postpartum period,[17] after caesarean delivery,[17] amniocentesis,[17] removal of placenta,[18] or with therapeutic abortion.[18] Other cases have been associated with abdominal trauma,[19] cervical suture removal,[20] ruptured uterus or intrapartum amnioinfusion.[20]

Prodromal symptoms in AFE are sudden chills, shivering, sweating, anxiety, and coughing followed by signs of respiratory distress, shock, cardiovascular collapse, and convulsions.[17]

Respiratory difficulty, evidenced by cyanosis, tachypnea, and bronchospasm, frequently culminates in fulminant pulmonary edema.

Hypoxemia explains the cyanosis, restlessness, convulsions, and coma. Reflex tachypnea results from the decreased arterial oxygen saturation and cardiovascular collapse, heralded by hypotension, tachycardia, and arrhythmia may end in cardiac arrest.

Convulsions may be an early manifestation of central nervous system involvement combined with cerebral ischemia and eventually may lead to coma and death. If the patient survives this initial episode, bleeding occurs secondary to disseminated intravascular coagulopathy and uterine atony. Common signs and symptoms of AFE are listed here [Table 1].

**Table 1:** Common signs and symptoms of amniotic fluid embolism

Hypotension*
Hypoxia*
DIC*
Altered mental status*
Seizure activity
Fever
Chills
Headache
Nausea
Vomiting
Evidence of fetal distress

* Present in 80% to 100% of affected women

## Diagnosis

The diagnosis of AFE is a clinical diagnosis and is one of exclusion. It is made on the basis of the clinical presentation. The initial signs may often be seen on an electrocardiogram (tachycardia with a right ventricular strain pattern and ST-T wave changes) and pulse oximetry may show sudden drop in oxygen saturation. This is followed by profound hypotension and cardiovascular collapse associated with severe respiratory distress. There is a subset of patients in whom severe hemorrhage with DIC may be the first sign. However, a definitive diagnosis is usually made by demonstration of amniotic fluid material in the maternal circulation and in the small arteries, arterioles, and capillaries of the pulmonary vessels.

In a living patient, diagnosis can be made by identification of lanugo or fetal hair and fetal squames in an aspirate of blood from the right heart.[21] Fetal squames have been recovered in the maternal sputum in some cases.[22]

Additional diagnostic tools for confirmation of amniotic fluid embolism suspected clinically include:

1. Chest X-ray: May show an enlarged right atrium and ventricle and prominent proximal pulmonary artery and pulmonary edema.
2. Lung scan: May demonstrate some areas of reduced radioactivity in the lung field.
3. Central venous pressure (CVP) with an initial rise due to pulmonary hypertension and eventually a profound drop due to severe hemorrhage.
4. Coagulation profile: Normally in pregnancy, blood coagulation factors increase. However, with amniotic fluid embolism, evidence of disseminated intravascular coagulation ensues with failure of blood to clot, decreased platelet count, decreased fibrinogen and afibrinogenemia, prolonged PT and PTT, and presence of fibrin degradation products.

## Differential Diagnosis

Any condition that presents as acute cardiopulmonary collapse or massive hemorrhage in the peripartum period must be systematically evaluated. The following entities be considered to differentiate AFE from the other causes.[22]

1. *Thrombotic pulmonary embolism*, is usually associated with chest pain. However, it generally occurs later in the postpartum period, and may occur with evidence of venous thrombosis in the lower limbs.[23]
2. *Air embolism*, is associated with chest pain, but an important differentiating factor from amniotic fluid embolism is the auscultation of a typical “water wheel” murmur over the pericardium.[21]
3. *Eclamptic convulsions and coma*, may resemble AFE, but the state of shock in AFE and the presence of hypertension, proteinuria, and edema in the eclamptic patient differentiate these two conditions.[24]
4. *Convulsion from toxic reaction to local anaesthetic drugs*, may be confounded with this syndrome. However the onset of symptoms and administration of the drug is an important differentiating factor.[24]
5. *Acute left heart failure*, is seen most commonly in pregnant patients with rheumatic heart disease and may simulate an AFE. The history of previous disease with ECG changes and cardiac murmur helps in the diagnosis.
6. *Cerebrovascular accident*, is distinguished from AFE by the absence of cyanosis, hypotension and pulmonary edema. Furthermore, examination of cerebrospinal fluid will help in the diagnosis.
7. *Aspiration of gastric contents* into the lungs causes cyanosis, tachycardia, hypotension, and pulmonary edema (similar to AFE). However, acid aspiration is usually seen in an unconscious patient with loss of the cough reflex or during induction or emergence from general anesthesia.[23]
8. *Hemorrhagic shock* in an obstetric patient, which is usually associated with ruptured uterus, uterine inversion, abruptio placenta, and placenta praevia, may lead to the erroneous diagnosis of AFE. A careful history and physical examination, absence of cyanosis and presence of low CVP with hemorrhagic shock should lead to the correct diagnosis.

## Laboratory Investigations

There are no specific laboratory tests that confirm the diagnosis, but some tests may support the diagnosis. Initial laboratory data should include:

1. An arterial blood gas to assess ventilation and the degree of hypoxemia;
2. Hematocrit will fall with associated hemorrhage;
3. White cell count may be elevated as an acute phase reactant;
4. Prolonged prothrombin and thromboplastin times with decreased fibrinogen levels due to accompanying DIC;
5. Cardiac enzymes and serum tryptase levels may be elevated;
6. Chest radiography may reveal pulmonary edema but is not diagnostic;
7. Echocardiography may demonstrate acute left heart failure, acute right heart failure or severe pulmonary hypertension.

Historically, it was believed that the amniotic fluid debris found by aspirating blood from the distal port of a pulmonary artery catheter was pathognomonic of the syndrome. These samples may have been contaminated by maternal squamous cells.[25,26] This test is more supportive of the diagnosis when squamous cells are found in large numbers, are coated with neutrophils, and/or if they are accompanied by other fetal debris.[27,28] Some investigators have used immunostaining of the antimucin monoclonal TKH-2 antibodies in the maternal serum and lung tissue.[29] TKH-2 reacts with meconium and mucin and stains the lung tissue in those with AFE. Another method described has been the measurement of zinc coproporphyrin, a component of amniotic fluid found in maternal serum, which is elevated in patients with AFE [Table 2].[30]

**Table 2:** Laboratory investigations

Non-specific	Specific
Complete blood count	Cervical history
Coagulation parameters including	
FDP, fibrinogen	
Arterial blood gases	Serum tryptase
Chest X-ray	Serum sialye
Electrocardiogram	Tn antigen
Echocardiogram	Zinc coproporphyrin

The diagnosis is confirmed postmortem by the presence of fetal squamous cells and other elements of fetal debris in the pulmonary vessels.

## Management

To prevent amniotic fluid embolism, trauma to the uterus must be avoided during maneuvers such as insertion of a pressure catheter or rupture of membranes. Incision of the placenta during caesarean delivery should also be avoided if possible.[7] Since, one of the most frequent predisposing factors is considered to be tumultuous labor that may occur naturally, excessively strong and frequent uterine contractions should be controlled by administration of intravenous β-adrenergic drugs[7] or magnesium sulphate.[31,32] Also, oxytocic drugs, which can precipitate excessive tetanic uterine contractions must be used appropriately and judiciously.

The key factors in the management of AFE are early recognition, prompt resuscitation, and delivery of the fetus. The input of consultants (anesthesiologist, obstetricians, hematologists, intensivists) must be enlisted early.

Monitoring of the patient with suspected amniotic fluid embolism includes continuous cardiac and respiratory monitoring with pulse oximetry or with an end-tidal CO_2_ monitor. If the patient has not yet delivered, a continuous fetal monitoring device should be placed. Blood pressure monitoring is performed with frequent serial noninvasive measurements or by continuous blood pressure readings. Obtaining intravenous access is of paramount importance. Insertion of a central venous catheter or a pulmonary artery catheter may guide the hemodynamic management of the patient, but insertion of catheters should never delay appropriate and expeditious treatment.

One difficulty in the initial treatment of amniotic fluid embolism is the rapidity at which signs and symptoms can occur; that should prompt the clinician to be vigilant and ready to implement therapy as needed. The time from delivery to the onset of symptoms has ranged from 15 to 45 minutes and the time from collapse to death has been reported as 1-7 hours.[33]

The management of amniotic fluid embolism is supportive and focuses initially on rapid maternal cardiopulmonary stabilization and adequate oxygenation to the vital organs [Table 3]. The majority of patients need to be managed in an intensive care unit after initial stabilization. The most important goal of therapy is to prevent additional hypoxia and end-organ hypoperfusion and organ system failure. Oxygen is given immediately to prevent the initial acute hypoxia seen in AFE and to prevent subsequent severe neurologic impairment seen in a significant number of survivors. Irrespective of whether oxygen is delivered by face mask, bag-valve mask, or endotracheal intubation, the immediate therapy is to deliver oxygen expeditiously. Positive end expiratory pressure (PEEP) is often needed to improve oxygenation. Fluid resuscitation is imperative to counteract hypotension and hemodynamic instability. Transthoracic or transesophageal echocardiography may guide fluid therapy with evaluation of left ventricular filling.[34] An arterial line and pulmonary catheter may also help to guide therapy. For refractory hypotension, vasopressor therapy is indicated. Epinephrine may be the first-line agent of choice, as it is used in other anaphylactoid reactions. Dopamine or noradrenaline may be ideal agents because of the additional β-adrenergic effects, which improve cardiac function in addition to the α-adrenergic vasoconstrictor effects. Inotropic support with dobutamine or milrinone may be needed. Therapeutic heparinization to minimize consumption coagulopathies remains controversial. The mortality from DIC may be great as 75%, in spite of optimal therapy. DIC with hemorrhage is treated based on the degree of hemorrhage with RBC and platelets transfusions, if thrombocytopenia is present. Specific coagulation laboratory abnormalities are treated with fresh frozen plasma, cryoprecipitate, fibrinogen, and/or factor replacement. Several newer therapies have been described in the treatment of AFE, although they only have been reported anecdotally and in very small case reports[35–43] [Table 4].

**Table 3:** Management of amniotic fluid embolism

Symptomatic (depends on severity)
Goals and treatment
Maintenance of oxygenation
Supplemental O_2_
Tracheal intubation
Ventilation
Circulatory support
*CPR protocol*
Delivery of fetus
Volume
Inotropes
After load reduction
Correction of the coagulopathy
Fresh frozen plasma
Packed RBC
Platelets
Cryoprecipitate
Possible additional measures
High dose corticosteroids
Adrenaline
Cardiopulmonary bypass
Nitric oxide
Inhaled prostacyclin

**Table 4:** Newer strategies in the management of amniotic fluid embolism

Intra aortic balloon counterpulsation[29]
Extracorporeal membrane oxygenation[29]
Cardiopulmonary bypass[30]
Plasma exchange transfusion[31,32]
Uterine artery embolization[33]
Continuous hemofiltration[32]
Cell-salvage combined with blood filtration[34]
Serum Protease inhibitors[35]
Inhaled nitric oxide[35]
Inhaled prostacyclin[35]
High dose corticosteroids[35]

Fetal condition often deteriorates in women in whom AFE develops during pregnancy. After 24 weeks' gestation, continuous and watchful monitoring of fetal heart rate is essential. Profound fetal acidemia often develops with maternal collapse, and early delivery may improve the fetal outcome. The performance of caesarean delivery in an unstable patient is fraught with pitfalls, therefore, management must be individualized. Although the primary responsibility of the physician is to ensure the health and life of the mother, intervention on behalf of the fetus is appropriate in certain instances.[8] Uterine tone should be maintained in the usual way using oxytocin, ergometrine, and prostaglandins such as carboprost and misoprost as indicated. Bimanual uterine massage and uterine packing may help to reduce blood loss.

If the patient with AFE sustains cardiac arrest, her chances of a good neurologically outcome is poor. Delivery may actually improve the likelihood of survival. Relieving uterine compression of the inferior vena cava may improve cardiac output by increasing preload. Therefore, perimortem caesarean delivery is recommended in pregnant woman with AFE and cardiac arrest.

## Prognosis

Survival after AFE has improved significantly with early recognition of this syndrome and prompt and early resuscitative measures.

The decrease in the mortality rate results solely from early diagnosis and prompt treatment rather than prevention of the syndrome, since the cause is unknown. Those women who survive long enough to be transferred to the ICU have a better chance of survival. Although mortality rates have declined, morbidity remains high with severe sequelae, particularly neurologic impairment.

## Family Support

Because the maternal and fetal mortality rates are so high, it is important to support the patient's family members. When the mother and infant are gravely ill, keeping their family members well informed and allowing as much access to the loved ones as possible are important.

## Conclusion

Amniotic fluid embolism syndrome is an infrequent, unpredictable, and catastrophic complication of pregnancy. It is virtually impossible to predict which patients are at risk for AFE. Diagnosis must be based on a spectrum of clinical signs and symptoms and by exclusion of other causes. Most cases of AFE are associated with dismal maternal and fetal outcomes, regardless of the quality of care rendered. Improved understanding of the pathophysiology of AFE may lead to the development of preventive measures and more effective and specific treatment. Although there are many new developments with respect to the understanding of the disease, amniotic fluid embolism continues to be a catastrophic illness requiring a high index of suspicion, a multidisciplinary approach and rapid resuscitation efforts in order to have a desirable clinical outcome.
